# Deep learning–based radiomic nomograms for predicting *Ki67* expression in prostate cancer

**DOI:** 10.1186/s12885-023-11130-8

**Published:** 2023-07-08

**Authors:** Shuitang Deng, Jingfeng Ding, Hui Wang, Guoqun Mao, Jing Sun, Jinwen Hu, Xiandi Zhu, Yougen Cheng, Genghuan Ni, Weiqun Ao

**Affiliations:** 1 Department of Radiology, Tongde Hospital of Zhejiang Province, No. 234 Gucui Road, Zhejiang Province 310012 Hangzhou, China; 2 Department of Radiology, Shanghai Putuo District People’s Hospital, Shanghai, China; 3 Department of Radiology, The Second Affiliated Hospital of Jiaxing University, Jiaxing, Zhejiang Province China

**Keywords:** Prostate cancer, Ki67, Deep learning, Prognosis

## Abstract

**Background:**

To explore the value of a multiparametric magnetic resonance imaging (MRI)-based deep learning model for the preoperative prediction of *Ki67* expression in prostate cancer (PCa).

**Materials:**

The data of 229 patients with PCa from two centers were retrospectively analyzed and divided into training, internal validation, and external validation sets. Deep learning features were extracted and selected from each patient’s prostate multiparametric MRI (diffusion-weighted imaging, T2-weighted imaging, and contrast-enhanced T1-weighted imaging sequences) data to establish a deep radiomic signature and construct models for the preoperative prediction of *Ki67* expression. Independent predictive risk factors were identified and incorporated into a clinical model, and the clinical and deep learning models were combined to obtain a joint model. The predictive performance of multiple deep-learning models was then evaluated.

**Results:**

Seven prediction models were constructed: one clinical model, three deep learning models (the DLRS-Resnet, DLRS-Inception, and DLRS-Densenet models), and three joint models (the Nomogram-Resnet, Nomogram-Inception, and Nomogram-Densenet models). The areas under the curve (AUCs) of the clinical model in the testing, internal validation, and external validation sets were 0.794, 0.711, and 0.75, respectively. The AUCs of the deep models and joint models ranged from 0.939 to 0.993. The DeLong test revealed that the predictive performance of the deep learning models and the joint models was superior to that of the clinical model (*p* < 0.01). The predictive performance of the DLRS-Resnet model was inferior to that of the Nomogram-Resnet model (*p* < 0.01), whereas the predictive performance of the remaining deep learning models and joint models did not differ significantly.

**Conclusion:**

The multiple easy-to-use deep learning–based models for predicting *Ki67* expression in PCa developed in this study can help physicians obtain more detailed prognostic data before a patient undergoes surgery.

## Background

Prostate cancer (PCa) is the most common malignancy and is responsible for the second-highest rate of cancer-related mortality in men [1, 2]. Advanced PCa has a high rate of bone metastasis, which severely affects the survival of patients. Therefore, early diagnosis of PCa is crucial [3]. Multiparametric MRI is considered one of the most effective imaging methods for the diagnosis of PCa and plays a key role in the diagnosis, staging, treatment evaluation, and prognosis of PCa [4, 5]. Adequate preoperative assessment of PCa can help physicians formulate personalized treatment and follow-up plans and is beneficial to patients’ long-term prognoses.

*Ki67* is a marker gene of tumor cell proliferation and is involved in cell anabolism. It is expressed throughout the cell cycle, except for the G0 phase. High *Ki67* expression implies active cell proliferation; the *Ki67* index can reflect the proliferation capacity of tumor cells [6, 7]. High *Ki67* expression in PCa is a poor prognostic factor and is correlated with overall survival, disease-free survival, and distant metastasis [8, 9]. The existing methods for detecting *Ki67* expression levels are invasive, complex, barely reproducible, and susceptible to subjective influence [10, 11]. Therefore, researchers must develop noninvasive, simple, and reproducible methods for detecting *Ki67* expression in patients with PCa. The results of the present study may facilitate the formulation of more precise treatment plans for patients with PCa.

As the core of artificial intelligence, deep learning algorithms have achieved amazing accuracy in image recognition and object detection in recent years [12, 13]. Convolutional neural networks (CNNs), a representative class of deep learning algorithms which comprise convolutional, pooling, excitation, and fully connected layers, have been applied extensively in radiomics. Models constructed using CNNs can automatically learn to extract and select image features used to make predictions; such models facilitate deep mining of image information [14] and have extensive application prospects.

Regarding noninvasive preoperative prediction of *Ki67* expression in PCa, Zhang et al. [15] used quantitative parameters of dynamic contrast-enhanced (DCE) MRI to preoperatively predict *Ki67* expression. Among the quantitative parameters examined therein, K^trans^ achieved the highest predictive performance with an AUC value of 0.826, whereas K^ep^ yielded an AUC of 0.784. Fan et al. [10] used three MRI sequences (T2-weighted imaging [T2WI], diffusion-weighted imaging [DWI], and DCE MRI) to construct various radiomics prediction models to predict *Ki67* expression in PCa. The random forest model achieved the highest performance. The models developed in the aforementioned studies used quantitative parameters of functional MRI or handcrafted radiomics to predict *Ki67* expression in PCa; however, these models were not subjected to external validation, and their stability and reliability therefore remain questionable. To date, no studies have evaluated the use of multiple deep learning models for the prediction of *Ki67* expression. Therefore, in the present study, three CNN-based deep learning models (Resnet101, Inception_v3, and Densenet121) for preoperatively predicting *Ki67* expression in PCa were constructed. The models were validated using internal and external data sets and may therefore serve as reliable tools in the development of personalized preoperative treatment plans.

## Methods

### Patients

**Fig. 1:**
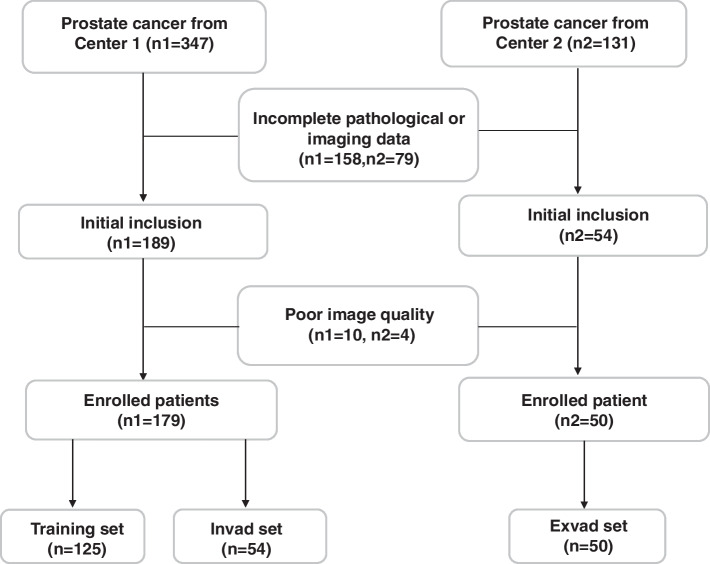
Flow diagram of enrolled patients in this study

This study was conducted in accordance with the Declaration of Helsinki in 1964 and approved by the Institutional Review Board (IRB) of Tongde Hospital of Zhejiang Province (2022-234 K) and Shanghai Putuo District People’s Hospital (2022-7). The need for informed consent for this retrospective study was waived. The data of 229 patients with PCa confirmed by pathology reports from January 2019 to December 2021 at two centers were collected. The patients were aged 44–94 years (mean: 75.5 ± 8.1 years). All the patients underwent regular T2WI, DWI, and enhanced MRI examinations. The inclusion criteria were as follows: (1) All patients’ MRI examinations were performed prior to puncture biopsy and surgery; (2) complete clinicopathological data; and (3) no antitumor treatment before the MRI examination. The exclusion criteria were as follows: (1) incomplete imaging and clinical data and (2) poor image quality that would hinder image analysis.(Fig. 1).

### Image analysis

All the patients received 3.0T MRI (Verio, Siemens, Germany) or 1.5T MRI (Avanto, Siemens, Germany) scanning equipped with 8-channel abdominal coil. Automatic gradient shimming was used to bias field correction. The selected sequences including axial T2WI, DWI, and contrast-enhanced T1WI (CE- T1). MRI scanning parameters were summarized in Table 1. The patients’ clinical and pathological data were recorded. Age, tumor location (peripheral, transitional zone or both involvement), long diameter (LD), short diameter (SD), ADC values, total prostate specific antigen (TPSA), free prostate specific antigen (FPSA), MRI-based TN staging (mrTN), M stage was assessed based on radiological (including X-ray plain film, CT, MRI and/or Emission CT bone scan) examination (rM), Ki67 expression, capsule invasion (CI), seminal vesicle invasion (SVI) and enhance mode (EMode). The cutoff value for *Ki67* expression was set to 10%, and the patients were divided into a low-expression group (< 10%) and high-expression group (≥ 10%) accordingly.

**Table 1:** MRI scanning parameters in two centers

Sequence	Parameters	Siemens 3.0T	Siemens 1.5T
T2WI	TR/TE,ms	4000/97	4120/97
	FOV,mm	200*200	200*200
	Thickness,mm	3.0	3.0
	Matrix	426*256	460*512
DWI	b value	0,800,1500	0,1000
	TR/TE,ms	9700/93	3439/95
	FOV,mm	256*256	250*250
	Thickness,mm	3.0	3.0
	Matrix	213*106	144*192
CE-T1	TR/TE,ms	5.1/1.7	7.64/2.77
	FOV,mm	256*256	234*250
	Thickness,mm	3.0	3.0
	Matrix	426*256	180*256

### Deep learning process

**Fig. 2:**
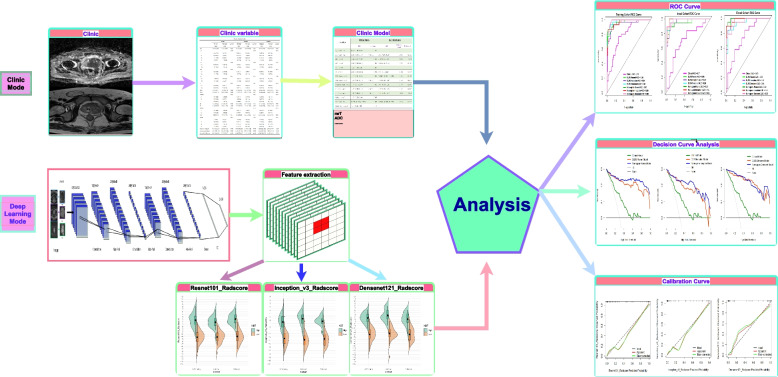
Flowchart of the study. First, model construction: Independent predictive factors of Ki67 expression in PCa were identified and incorporated into the clinical model. Three deep learning models were constructed based on different deep-learning neural network architectures (Resnet101, Inception_v3, and Densenet121), then deep learning signatures were calculated accordingly. Then the three deep learning models were combined with the selected clinical features (mrT and ADC values) to create three nomograms. Second, model evaluation: the ROC curves, nomogram plots, and calibration curves were used for model evaluation

Step 1 (Preparing the data sets): Python 3.9 (https://www.python.org/) and PyCharm Community Edition (https://pytorch.org/)  were used for data processing. The prostate MRI images from the 229 patients included in the data set comprised three MRI sequences (T2WI, DWI, and T1C). A total of 687 images in JPG format were processed. The regions of interest (ROI) of the lesions with the maximum cross-sectional image of prostate cancer lesions were selected and manually cut close to the edge of the prostate gland on each image into a 256 × 256 size as jpg format by an abdominal radiologist (10 years of experience) and confirmed by the pathologist (15 years of experience) based on the pathological results. Subsequently, the outlined images were confirmed by another abdominal radiologist with 20 years of experience. If their opinions conflicted, the conflicts were resolved through discussion until a consensus was reached. All the images were converted into jpg format and were resized to 256 × 256. To avoid data heterogeneity, all the images were normalized by the normalize [transforms. Normalize (mean, std)] function: x= (x - mean)/std. The data from Center 1 were divided into training and testing sets in a ratio of 7:3. The models were constructed using the training set and tested on the testing set and external data (Fig. 2).

Step 2 (Constructing the CNN): First, three models were constructed using different deep-learning neural network architectures (Resnet101, Inception_v3, and Densenet121). The details of three CNN models in present study are summarized as follow: (1) Resnet101 residual network structure: First 7 × 7 × 64 convolution is carried out, followed by 33 (3 + 4 + 23 + 3) building blocks. Each block includes 3 layers, so the blocks altogether contain 99 (33 × 3) layers. With the last FC layer (fully connected layer) included, the network finally comprises a total of 101 (1 + 99 + 1) layers. The 101-layer network only refers to the convolution layer or the full connection layer, however the second-activation layer or Pooling layer is not included. (2) DenseNet121 dense convolutional network structure: The DenseNet network structure mainly consists of three core structures, namely, DenseLayer (the most basic atomic unit of the model, which provides the initial feature extraction), DenseBlock (the basic unit of the model’s dense connection) and Transition (the transition unit among different dense connections for convolutional and pooling layers, which is used to integrate the learned features and reduce the size of the feature map). The model can be built through splicing and layer classification of the above structures. The DenseBlock of DenseNet-121 containing six BottleNecks that are linked in series passes through the convolutional layer and the pooling layer, and then allows for output at the fully connected layer. (3) InceptionV3 network structure: Inception-v3 model consists of 46 layers in total and 11 Inception modules, which integrates different convolutional layers in parallel, mainly by input matrix, intermediate structure and output matrix.

The prostate MRI data were input into the models. The input data first passed through the convolution layer, in which parameters such as the numbers of input channels (In channel) and output channels (Out channel), convolution kernel size, and convolution stride were set. The data then passed through the max-pooling layer. The data repeatedly passed through the convolution layer and max-pooling layer before being subjected to dimensionality reduction and flattening. The deep learning features were extracted from the last fully connected layer. Preliminary screening of the deep learning features was performed using the minimum redundancy maximum correlation (mRMR) method; thereafter, the least absolute shrinkage and selection operator (LASSO) method with 10-fold cross-validation, was used to further reduce the dimensions and select the strongest relevant features to construct a model.

Step 3 (Training and testing): First, the parameters (i.e., number of iterations and rounds) for CNN training were set. Second, the loss function and optimizer model were defined. Finally, the test data set was input into the CNN model for testing, and evaluation indicators such as sensitivity, specificity, and accuracy were calculated.

### Statistical analysis

Statistical analysis was performed using R software version 3.6.1 (R Core Team [2019], http:www.r-project.rog). Normally distributed continuous variables are herein presented as means ± standard deviations, and categorical variables are presented as frequencies and percentages. A *t* test and rank-sum test were used to analyze data with and without normal distributions, respectively; a chi-square test was used to analyze count data. Univariate and multivariate analyses were performed on the clinical variables in the training set, and independent predictors were selected and incorporated into a clinical model. Receiver operating characteristic (ROC) curves were used to evaluate the predictive performance of the models. The DeLong test was used to perform pairwise comparisons of the ROC curves of each model. The calibration curve was used to test the degree of model calibration, and the decision curve was used to analyze the net clinical benefit of each model. All the *p* values were two-tailed, and statistical significance was defined as a *p* value of < 0.05.

## Results

### Patient clinical features

A total of 229 patients were pathologically confirmed PCa. Among them, 214 patients underwent prostate cancer puncture biopsy, and 15 patients underwent radical prostatectomy. Of the patients in the training, internal validation, and external validation sets, 31 (24.8%) and 19 (35.2%), and 19 (38%), respectively, had high *Ki67* expression. In the training set, age, number of lesions and EMode had no statistical difference (*P* > 0.05), while mrT, mrN, mrM, TPSA, Location, LD, SD and ADC values ​​of the high- and low-expression groups differed significantly (*p* < 0.05). A comparison of the clinical features of the patients in the training, internal validation, and external validation sets is presented in Table 2. According to European Association of Urology-European Association of Nuclear Medicine-European Society for Radiotherapy and Oncology-European Society of Urogenital Radiology-International Society of Geriatric Oncology (EAU-EANM-ESTRO-ESUR-SIOG) guidelines on prostate cancer, Gleason score of 7 was used as the critical value to divide patients into low-risk (< 7) and high-risk (≥ 7) groups [16]. There were 65 cases of low-risk and 164 cases of high-risk in this study.

**Table 2:** Comparison of Clinical and imaging features with different Ki67 status in three sets

	**A Train**			**B Invad**			**C Exvad**		
	**Ki67(low)**	**Ki67(high)**	**p.overall**	**Ki67(low)**	**Ki67(high)**	**p.overall**	**Ki67(low)**	**Ki67(high)**	**p.overall**
	***N=94***	***N=31***		***N=35***	***N=19***		***N=31***	***N=19***	
Age	76.0 [72.0;82.0]	74.0 [67.5;82.0]	0.386	77.0 [69.0;81.0]	76.0 [68.0;83.5]	0.863	73.0 [69.0;78.5]	76.0 [71.5;80.0]	0.280
mrT			<0.001			0.069			0.614
I	43 (45.7%)	3 (9.68%)		16 (45.7%)	3 (15.8%)		15 (48.4%)	6 (31.6%)	
II	31 (33.0%)	9 (29.0%)		12 (34.3%)	7 (36.8%)		10 (32.3%)	9 (47.4%)	
III	11 (11.7%)	8 (25.8%)		5 (14.3%)	5 (26.3%)		5 (16.1%)	3 (15.8%)	
IV	9 (9.57%)	11 (35.5%)		2 (5.71%)	4 (21.1%)		1 (3.23%)	1 (5.26%)	
mrN			0.026			0.087			0.273
No	80 (85.1%)	20 (64.5%)		30 (85.7%)	12 (63.2%)		27 (87.1%)	14 (73.7%)	
Yes	14 (14.9%)	11 (35.5%)		5 (14.3%)	7 (36.8%)		4 (12.9%)	5 (26.3%)	
rM			0.006			0.169			0.549
No	83 (88.3%)	20 (64.5%)		33 (94.3%)	15 (78.9%)		30 (96.8%)	17 (89.5%)	
Yes	11 (11.7%)	11 (35.5%)		2 (5.71%)	4 (21.1%)		1 (3.23%)	2 (10.5%)	
SVI			0.877			0.297			1.000
No	76 (80.9%)	24 (77.4%)		30 (85.7%)	14 (73.7%)		28 (90.3%)	17 (89.5%)	
Yes	18 (19.1%)	7 (22.6%)		5 (14.3%)	5 (26.3%)		3 (9.68%)	2 (10.5%)	
CI			0.005			0.364			0.011
No	44 (46.8%)	5 (16.1%)		15 (42.9%)	5 (26.3%)		21 (67.7%)	5 (26.3%)	
Yes	50 (53.2%)	26 (83.9%)		20 (57.1%)	14 (73.7%)		10 (32.3%)	14 (73.7%)	
TPSA	14.0 [9.64;31.1]	41.4 [23.3;100]	<0.001	13.9 [8.60;23.4]	32.0 [15.7;100]	0.005	14.9 [8.82;46.7]	27.3 [12.9;45.1]	0.484
FPSA	1.77 [1.03;3.84]	6.78 [2.68;30.0]	<0.001	1.10 [0.77;2.75]	3.96 [2.17;12.8]	0.001	1.67 [0.86;4.48]	2.43 [1.11;5.22]	0.660
Location:			0.005			0.213			0.458
A	46 (48.9%)	10 (32.3%)		13 (37.1%)	3 (15.8%)		9 (29.0%)	4 (21.1%)	
B	20 (21.3%)	2 (6.45%)		8 (22.9%)	4 (21.1%)		10 (32.3%)	4 (21.1%)	
C	28 (29.8%)	19 (61.3%)		14 (40.0%)	12 (63.2%)		12 (38.7%)	11 (57.9%)	
LD	17.3 [11.8;22.2]	27.0 [20.7;39.7]	<0.001	15.4 [12.1;26.2]	23.7 [14.8;30.9]	0.150	16.1 [10.4;26.0]	22.1 [19.6;27.6]	0.084
SD	11.6 [7.88;15.7]	19.2 [14.1;23.9]	<0.001	10.9 [7.50;13.9]	14.7 [10.1;21.0]	0.025	10.3 [6.80;16.4]	13.8 [11.4;19.6]	0.101
Number:			0.639			0.194			0.759
U	46 (48.9%)	13 (41.9%)		18 (51.4%)	14 (73.7%)		19 (61.3%)	10 (52.6%)	
M	48 (51.1%)	18 (58.1%)		17 (48.6%)	5 (26.3%)		12 (38.7%)	9 (47.4%)	
ADC	580 [509;695]	521 [495;580]	0.007	578 [500;694]	557 [504;632]	0.556	650 [600;747]	583 [537;626]	0.002
EMode:			0.514			1.000			0.457
I	1 (1.06%)	0 (0.00%)		0 (0.00%)	0 (0.00%)		0 (0.00%)	0 (0.00%)	
II	34 (36.2%)	8 (25.8%)		13 (37.1%)	7 (36.8%)		7 (22.6%)	5 (26.3%)	
III	47 (50.0%)	16 (51.6%)		11 (31.4%)	6 (31.6%)		12 (38.7%)	10 (52.6%)	
IV	12 (12.8%)	7 (22.6%)		11 (31.4%)	6 (31.6%)		12 (38.7%)	4 (21.1%)	
DLRS-Resnet	-4.18 [-6.12;-2.37]	3.25 [0.74;4.23]	<0.001	-3.94 [-5.77;-2.58]	2.15 [0.34;3.28]	<0.001	-4.38 [-5.83;-1.85]	2.88 [1.53;4.67]	<0.001
DLRS-Inception	-3.46 [-5.00;-1.99]	1.60 [-0.16;3.14]	<0.001	-3.77 [-5.39;-2.65]	2.96 [1.07;3.63]	<0.001	-3.57 [-5.44;-2.18]	1.60 [1.01;2.48]	<0.001
DLRS-Densenet	-4.60 [-6.60;-2.66]	2.33 [0.89;4.27]	<0.001	-4.68 [-5.87;-2.47]	3.13 [2.14;5.99]	<0.001	-4.80 [-7.38;-2.71]	2.18 [0.48;4.51]	<0.001

*mrTN* MRI-based TN staging, *rM *M stage based on radiological examination, *Location* A = peripheral, B = transitional zone, C = both involvement, *LD* Long diameter, *SD* Short diameter, *CI* Capsule invasion, *TPSA* Total prostate specific antigen, *FPSA* Free prostate specific antigen, EMode I = no enhancement; II = progressive enhancement; III = fast-in and fast-out enhancement; IV: fast-in and slow-out enhancement pattern

### Construction and predictive performance of clinical model

**Fig. 3:**
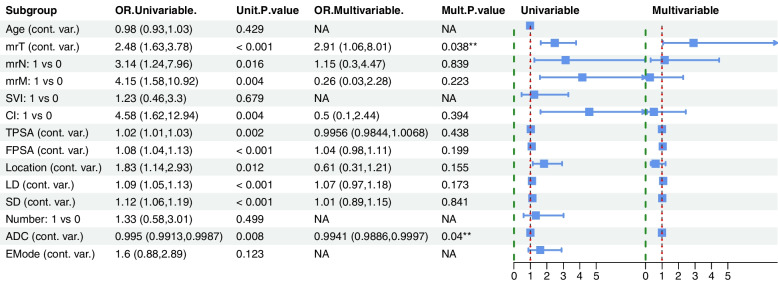
Clinical model construction. According to the results of univariate and multivariate logistic regression analysis, mrT and ADC values were identified as independent predictors of *Ki67* expression and were incorporated into the clinical model

In this study, KI67 expression level was considered as the dependent variable, fourteen Clinical and MRI indicators (including Age, mrT, mrN, rM, SVI, CI, TPSA, FPSA, Location, LD, SD, Number of lesions, ADC and EMode) of prostate diseases as independent variables. All the variables of interest were included in univariate and multivariate logistic regressions. Among the variables, mrT (OR = 0.91, *P* = 0.038) and ADC (OR = 0.99, *P* = 0.04) were identified as independent predictive factors of *Ki67* expression in PCa and were incorporated into the clinical model (Fig. 3). The AUCs of the clinical model in predicting *Ki67* expression in PCa were 0.794, 0.711, and 0.75 in the training, internal validation, and external validation sets, respectively.

### Predictive performance of deep learning models

**Fig. 4:**
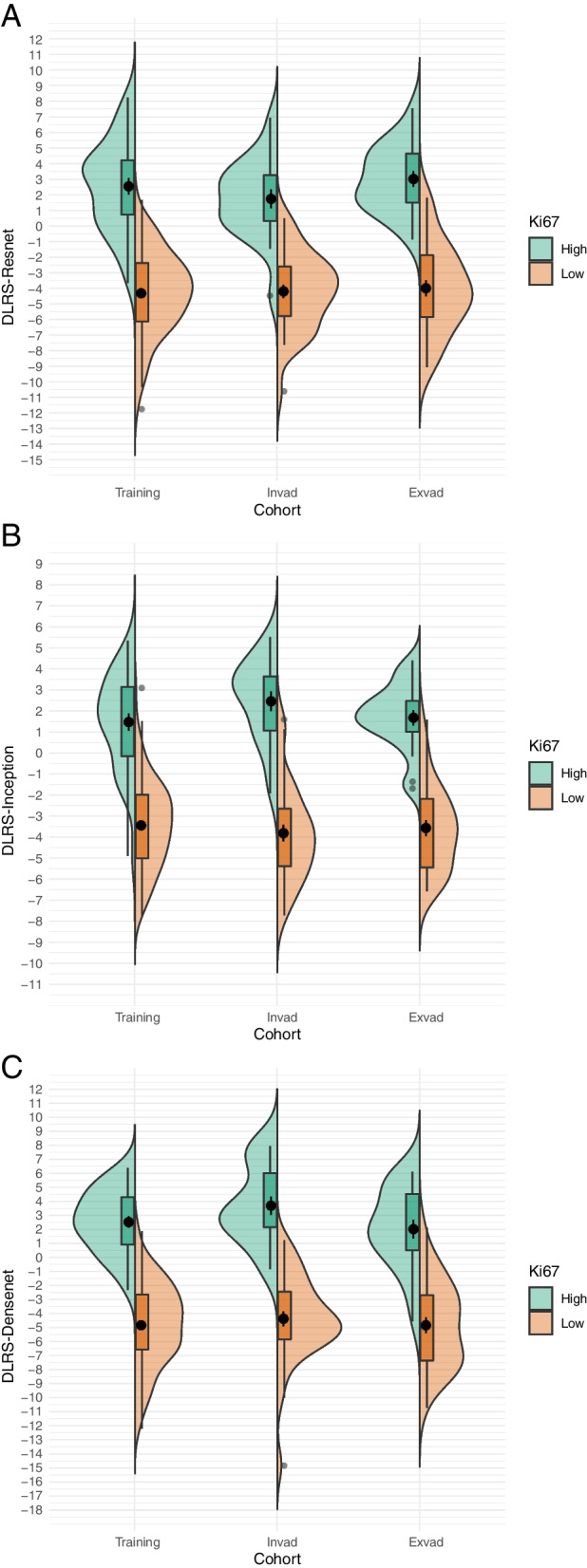
Distribution of radscores of DLRS- Resnet (**a**), DLRS- Inception (**b**) and DLRS- Densenet (**c**) between patients with high and low *Ki67* expression in the testing, internal validation, and external validation sets

The three models constructed in the present study (Resnet101, Inception_v3, and Densenet121) extracted 6144, 6144, and 3000 deep learning features, respectively. To avoid model overfitting due to feature redundancy, mRMR and Lasso regression were performed to filter out all the features with a high degree of multicollinearity. Finally, 43 (T2WI:17、DWI:13,CE-T1WI:13), 39 (T2WI:15、DWI:11,CE-T1WI:12), and 78 (T2WI:30、DWI:22,CE-T1WI:26) features related to *Ki67* expression were selected from among the features extracted by the Resnet101, Inception_v3, and Densenet121 models, respectively. Three deep learning image tagging models (the DLRS-Resnet, DLRS-Inception, and DLRS-Densenet models) were constructed using the Cox proportional-hazards model. According to the Youden’s index values in the training set, the optimal cutoff values for the DLRS models were determined to be 0.272, 0.192, and 0.285, respectively. In the training, internal validation, and external validation sets, the DLRS of the high- and low-expression groups differed significantly (*p* < 0.001). Figure 4 illustrates the DLRS distribution. The AUCs of DLRS-Resnet in predicting *Ki67* expression in PCa in the training, internal validation, and external validation sets were 0.961, 0.95, and 0.976, respectively; the corresponding AUCs of DLRS-Inception were 0.939, 0.97, and 0.973, respectively; and the corresponding AUCs of DLRS-Densenet were 0.98, 0.983, and 0.944, respectively (Table 3).

**Table 3:** Diagnostic efficiency in different models

	ROC	AUC	ACC	SEN	SPE	PPV	NPV
TRAIN	Clinic	0.794	0.744	0.774	0.734	0.49	0.908
	DLRS-Resnet	0.961	0.896	0.903	0.894	0.737	0.966
	DLRS-Inception	0.939	0.872	0.935	0.851	0.674	0.976
	DLRS-Densenet	0.98	0.928	0.935	0.926	0.806	0.978
	Nomogram-Resnet	0.975	0.952	0.871	0.979	0.931	0.958
	Nomogram-Inception	0.962	0.944	0.871	0.968	0.9	0.958
	Nomogram-Densenet	0.983	0.92	1	0.894	0.756	1
INVAD	Clinic	0.711	0.685	0.737	0.657	0.538	0.821
	DLRS-Resnet	0.95	0.907	0.895	0.914	0.85	0.941
	DLRS-Inception	0.97	0.926	0.947	0.914	0.857	0.97
	DLRS-Densenet	0.983	0.907	1	0.857	0.792	1
	Nomogram-Resnet	0.958	0.926	0.895	0.943	0.895	0.943
	Nomogram-Inception	0.988	0.963	1	0.943	0.905	1
	Nomogram-Densenet	0.986	0.963	0.895	1	1	0.946
EXVAD	Clinic	0.75	0.68	0.842	0.581	0.552	0.857
	DLRS-Resnet	0.976	0.94	1	0.903	0.864	1
	DLRS-Inception	0.973	0.92	1	0.871	0.826	1
	DLRS-Densenet	0.944	0.9	0.842	0.935	0.889	0.906
	Nomogram-Resnet	0.993	0.96	0.947	0.968	0.947	0.968
	Nomogram-Inception	0.983	0.96	0.895	1	1	0.939
	Nomogram-Densenet	0.952	0.88	0.895	0.871	0.81	0.931

### Nomogram construction and predictive performance

**Fig. 5:**
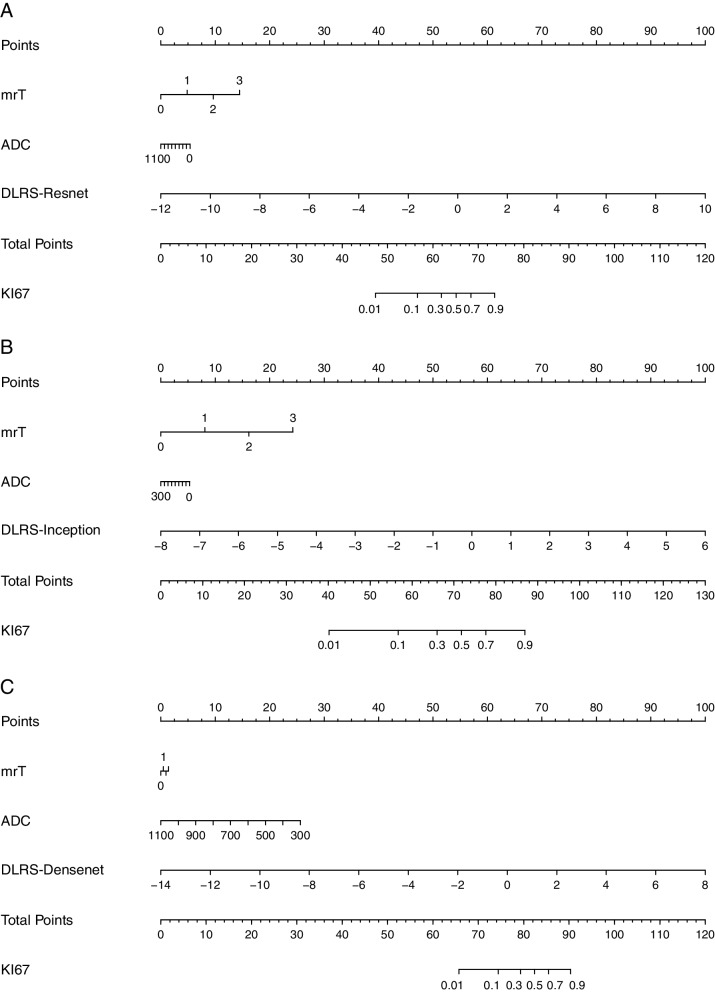
Incorporation of mrT, ADC, and radscores into the array plot for predicting *Ki67* expression in PCa. Nomogram-Resnet (**a**), Nomogram-Inception (**b**), and Nomogram-Densenet (**c**)

**Fig. 6:**
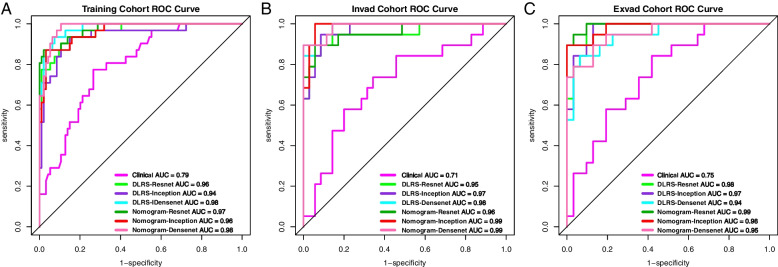
AUC curves of models in the testing (**a**), internal validation (**b**), and external validation sets (**c**)

The three deep learning models were combined with the selected clinical features (mrT and ADC values) to create three nomograms: Nomogram-Resnet, Nomogram-Inception, and Nomogram-Densenet (Fig. 5). The AUCs of Nomogram-Resnet in predicting *Ki67* expression in PCa in the testing, internal validation, and external validation sets were 0.975, 0.958, and 0.993, respectively; the corresponding AUCs of Nomogram-Inception were 0.962, 0.988, and 0.983, respectively; and the corresponding AUCs of Nomogram-Densenet were 0.983, 0.986, and 0.952, respectively. The AUC curves of each model are displayed in Fig. 6.

### Comparison of predictive performance of models

**Fig. 7:**
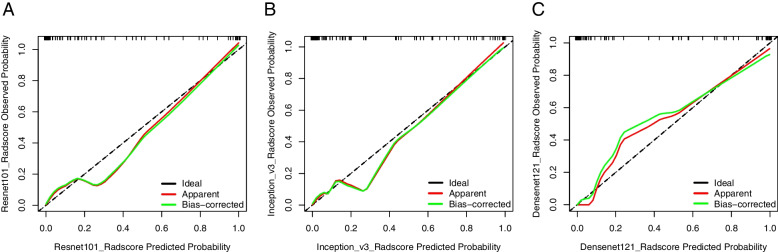
Calibration curve for deep learning models. Nomogram-Resnet (**a**), Nomogram-Inception (**b**), and Nomogram-Densenet (**c**). The *y* axis represents the observed probability; the *x* axis represents the nomogram-predicted probability; and the solid diagonal gray line represents a perfect prediction by an ideal model

**Fig. 8:**
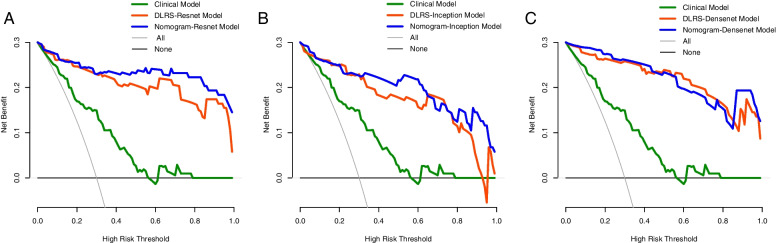
Results of decision curve analysis indicating that both the DLRS models and nomograms have a greater net benefit than does the clinical model in the testing (**a**), internal validation (**b**), and external validation sets (**c**)

**Fig. 9:**
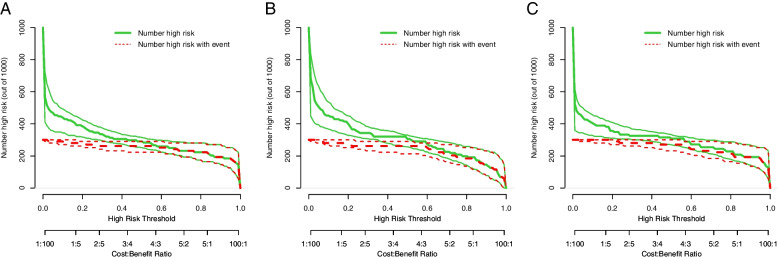
Clinical impact curves for 1000 random patients based on the integrated nomogram of the testing (**a**), internal validation (**b**), and external validation sets (**c**). The 95% confidence intervals calculated through bootstrapping are displayed on both sides of the ROC plot or clinical impact plot

**Fig. 10:**
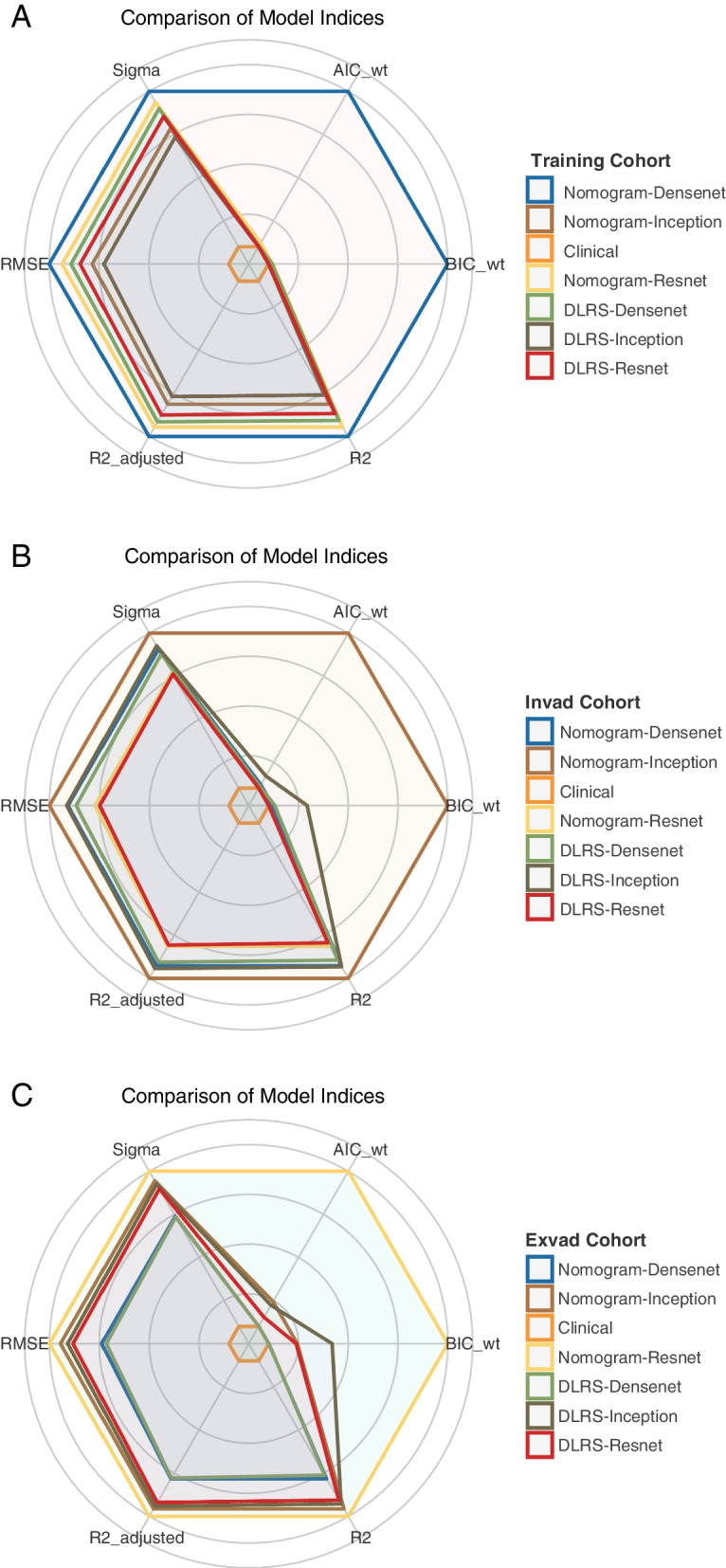
Radar diagrams are charts that display multidimensional model data. In the testing (**a**), internal validation (**b**), and external validation (**c**) sets, the Nomogram-Densenet Nomogram-Inception and Nomogram-Resnet models, respectively, had the largest area in the radar diagram, corresponding to the highest performance

The AUCs of the nomograms were higher than those of the clinical and DLRS models. The DeLong test revealed that the predictive performance of the DLRS models and nomograms was superior to that of the clinical model (*p* < 0.05), but the predictive performance of the DLRS models and nomograms did not differ significantly (Table 4). The calibration curves in Fig. 7 indicate that the *Ki-67* expression levels predicted using the nomograms were highly consistent with postoperative immunohistochemistry results. The closer the calibration curve is to the 45° line, the higher the recognition accuracy of the corresponding nomogram is. Decision curve analysis revealed that all the DLRS models and nomograms had a greater net benefit than did the clinical model (Fig. 8). Supplemental clinical impact curves were constructed to further illustrate the clinical value of the nomograms (Fig. 9). According to the radar chart (Fig. 10), the Nomogram-Densenet, Nomogram-Inception, and Nomogram-Resnet models exhibited the highest performance in the testing set, internal validation set, and external validation set, respectively.

**Table:** Delong test in different models

Model comparison	Z1	p1	Z2	p2	Z3	p3
Clinic vs DLRS-Resnet	-3.621	<0.001	-3.043	0.002	-3.054	<0.001
Clinic vs DLRS-Inception	-2.762	<0.001	-3.219	0.001	-3.086	0.002
Clinic vs DLRS-Densenet	-4.283	<0.001	-3.693	<0.001	-2.607	0.009
Clinic vs Nomogram-Resnet	-4.299	<0.001	-3.279	0.001	-3.548	<0.001
Clinic vs Nomogram-Inception	-4.008	<0.001	-3.628	<0.001	-3.418	<0.001
Clinic vs Nomogram-Densenet	-4.573	<0.001	-3.683	<0.001	-2.979	<0.001
DLRS-Resnet vs Nomogram-Resnet	-1.812	0.070	-0.926	0.354	-1.177	<0.001
DLRS-Inception vs Nomogram-Inception	-1.418	0.1561	-1.363	0.173	-0.767	0.443
DLRS-Densenet vs Nomogram-Densenet	-0.478	0.632	-0.404	0.686	-0.725	0.469
DLRS-Resnet vs DLRS-Densenet	-0.927	0.354	-0.930	0.353	0.978	0.328

P1= Training vs Invad, p2=p.Training vs Exvad, p3= Invad vs Exvad

## Discussion

To the best of our knowledge, this study is the first to use multisequence MRI to construct three deep learning models for the preoperative prediction of *Ki67* expression in PCa. The deep learning models were combined with independent predictive risk factors to create nomograms. Both the deep learning models and nomograms exhibited satisfactory predictive performance and were validated using internal and external data sets.

Previous studies have demonstrated that *Ki67* expression may be related to the prognosis of various tumors [17, 18]. Patients with PCa with high *Ki67* expression (> 10%) exhibit poor tumor differentiation and are at a higher risk of metastasis and recurrence. *Ki67* expression is a risk factor for poor prognosis in PCa [10, 19]. A study [6] showed that Ki67 upregulation has close relationship with PCa aggressiveness. Ki67 expression is associated with disease-free survival, biochemical recurrence and metastasis. Patients with high Ki67 expression are more likely (2.62 times) to develop biochemical recurrence than those with low Ki67 expression. Another study [20] suggests that Ki67 may provide additional prognostic information for Gleason reporting methods of PCa. However, accurately measuring *Ki67* expression through conventional imaging examinations is difficult. Radiomics can be used to mine digital information from medical images and reflect the heterogeneity of tumors [21].

Most radiomics studies conducted to date have involved the use of multilayer manual delineation for feature extraction [22]. Manual delineation is labor-intensive, time-consuming, and subject to subjective influence. The results of delineation and, in turn, the interpretation of features, vary between doctors. In the present study, an abdominal radiologist selected the ROIs of the lesions with the maximum diameter during image segmentation. Relative to manual delineation layer by layer, the method employed in this study has multiple advantages: the subsequent network is less affected by the accuracy of ROI segmentation, and the method itself is easy to use and can reduce physicians’ workloads. Thousands of deep learning features were extracted by the deep learning models to avoid model overfitting due to feature redundancy. Among the features, those with a high degree of multicollinearity were excluded, and those related to *Ki67* expression were selected. The selected features were strongly correlated with PCa tumor proliferation and invasion. The deep learning models DLRS-Resnet, DLRS-Inception, and DLRS-Densenet achieved high prediction accuracy, with AUCs ranging from 0.939 to 0.983.

The three deep learning models developed in this study had distinct characteristics. Resnet101 architecture is based on a 101-layer deep residual network, which enables it to overcome the vanishing gradient problem [23, 24] and achieve high classification accuracy. Inception architecture uses 1 × 1 convolution to reduce dimensionality and minimize computational complexity. The principle of decomposing a sparse matrix into a dense matrix can be used to accelerate convergence and achieve higher accuracy [25–27]. The number of output feature maps of each convolution layer in the dense block of Densenet is extremely small (< 100); the network is narrow and has few parameters. The connection makes the transmission of features and gradients more efficient, rendering the network easier to train [28, 29].

Regarding the noninvasive preoperative prediction of *Ki67* expression in tumors, although manual radiomics models based on image data to predict *Ki67* expression in the tumor cells of patients with bladder cancer, breast cancer, and lung cancer have been constructed in previous studies [30–32], few of these studies involved external validation. The models constructed in the present study therefore achieved higher prediction performance. In contrast to manual radiomics, deep learning models, like those constructed in this study, automatically learn from high-dimensional data through neural networks, which minimizes the need for feature engineering and saves time. In the present study, the introduction of deeper and more complex network structures required repeated adjustments in the models’ parameters to achieve optimal results. Internal and external validation demonstrated the reliability and robustness of the deep learning models.

The results of the present study indicate that the differences in MRI scanners and scanning parameters between centers exert no significant effect on the predictive performance of deep learning algorithms, which supports the reliability and robustness of the deep learning models. The AUCs of the nomograms constructed by combining clinical features and DLRS were optimal. Clinical data and medical images complement each other and can be combined to display tumor features from different angles [33]. Nomograms can be used to obtain more comprehensive information on tumor prognosis. Utilizing the proposed method enables physicians to develop more personalized and effective treatment strategies [34].

A study [35] adopted biparametric MRI (T2WI + ADC map) to characterize prostate cancer. Multiple integrated nomograms were established combining clinical variables, PI-RADS score and deep learning. The ClaD (clinical variables + PI-RADS score + deep learning) nomogram got the best predicting performance compared with deep learning model, DIN (clinical variables + deep learning) nomogram, and PIN (clinical variables + PI-RADS score) nomogram. The Prostate Imaging–Reporting and PI-RADS based on multiparameter MRI was widely applied and recognized in clinical practice. However, the subjective methods are often dependent on physician’s experience and expertise, differences exist among different individuals. By contrast, deep learning can obtain more features than the naked eye and reflect the heterogeneity of tumors. Similarity, Jing et al. [36] used PI-RADS and MRI radiomics signature (based on T2WI + DWI sequence) to construct nomogram for predicting clinically significant prostate cancer. They revealed that nomogram had better predictive performance than PI-RADS in both the training group, internal and external validation group. Compared with these studies, the present study has the following superiorities. First, multisequence single-layer segmented images were used for feature extraction and selection. Relative to other radiomic models, the proposed model is simpler and easier to use which saves much time on image segmentation and feature learning. Second, three deep learning prediction models with distinct characteristics were constructed, all of which exhibited satisfactory prediction performance. Third, the models were subjected to internal and external validation that determined to be stable and reliable.

This study has some limitations. First, the sample contained fewer patients with low *Ki67* expression than high *Ki67* expression, and the results must therefore be validated using a larger sample. Second, deep learning analysis process involved manual segmentation and was susceptible to subjective influences. Automatic segmentation technology will be further investigated in future follow-up studies. Finally, the degree to which immunohistochemical indicators reflect tumor features warrants further investigation.

## Conclusions

In this study, three easy-to-use DLRS models and radiomic nomograms for predicting *Ki67* expression in PCa were developed. The models exhibit strong predictive performance and may serve as a new noninvasive strategy for preoperative prediction of PCa prognosis.

## Data Availability

All data generated or analyzed during this study are included in this published article.

## Abbreviations

AUC: Area under the curve
T1C: Contrast enhancement T1WI
CI: Confidence interval
DCA: Decision curve analysis
DCE: Dynamic contrast-enhanced
DWI: Diffusion-weighted imaging
EMode: Enhance mode
Ex-valid: External validation
FPSA: Free prostate specific antigen
In-valid: Internal validation
LASSO: Least absolute shrinkage and selection order
LD: Long diameter
T2WI: T2-weighted imaging
in-valid: Internal validation
PCa: Prostate cancer
ROC: Receiver operating characteristic
SD: Short diameter
SVI: Seminal vesicle invasion
TPSA: Total prostate specific antigen
